# Stress, Environment and Early Psychosis

**DOI:** 10.2174/1570159X21666230817153631

**Published:** 2023-08-18

**Authors:** Lida-Alkisti Xenaki, Stefanos Dimitrakopoulos, Mirjana Selakovic, Nikos Stefanis

**Affiliations:** 1 First Department of Psychiatry, School of Medicine, Eginition Hospital, National and Kapodistrian University of Athens, 72 Vas. Sophias Ave., Athens, 115 28, Greece

**Keywords:** Psychosocial stressors, hypothalamic-pituitary-adrenal, first-episode psychosis, stress, environment, psychosis onset

## Abstract

Existing literature provides extended evidence of the close relationship between stress dysregulation, environmental insults, and psychosis onset. Early stress can sensitize genetically vulnerable individuals to future stress, modifying their risk for developing psychotic phenomena. Neurobiological substrate of the aberrant stress response to hypothalamic-pituitary-adrenal axis dysregulation, disrupted inflammation processes, oxidative stress increase, gut dysbiosis, and altered brain signaling, provides mechanistic links between environmental risk factors and the development of psychotic symptoms. Early-life and later-life exposures may act directly, accumulatively, and repeatedly during critical neurodevelopmental time windows. Environmental hazards, such as pre- and perinatal complications, traumatic experiences, psychosocial stressors, and cannabis use might negatively intervene with brain developmental trajectories and disturb the balance of important stress systems, which act together with recent life events to push the individual over the threshold for the manifestation of psychosis. The current review presents the dynamic and complex relationship between stress, environment, and psychosis onset, attempting to provide an insight into potentially modifiable factors, enhancing resilience and possibly influencing individual psychosis liability.

## 1 INTRODUCTION

The trailhead of psychotic phenomena has been the subject of long-enduring scientific research. Psychosis spectrum disorders range from severe, long-lasting, and debilitating diseases to short-lived, sub-clinical psychotic experiences in non-clinical populations. They are considered to have a substantial genetic component. However, despite elaborate genome-wide association studies (GWAS), which have reinforced our understanding of the genetic structure of schizophrenia, the polygenic susceptibility loci identified to date explain only a minority of the variance in risk profiles, underlining the multifactorial nature of the disease [1], and rendering the investigation towards environmental factors essential. Nurture determinants appear to explain the bulk of variance for mental disorders [2], and it is argued that genetic influence may be of limited explanatory power unless viewed in the context of interaction with socioenvironmental effects [3].

Umbrella reviews indicate that a number of environmental risk factors, such as childhood adversity, cannabis use, urbanicity, social defeat (*i.e*., migration status, ethnical minority), obstetric complications, and season of birth are associated with the emergence of psychosis [4, 5]. Indeed, meta-analytic studies suggest that over 30% of psychosis cases could be attributable to early environmental adversities [6, 7], and it has been found that compared to genetic risk, exposure of individuals to deleterious environmental exposures generally exhibits higher odds ratios than common genetic variant discerned through GWAS [8]. Some of these environmental factors might be interconnected, having cumulative/additive effects and/or sharing common pathogenic pathways [9]. Furthermore, the severity of the exposure is correlated with an unfavorable clinical outcome [10] in terms of the intensity of symptoms, resistance to treatment, and recurrence of disease [11, 12]. The association of environmental stressors with psychotic phenomena has been reported in both general and clinical population subjects [6, 13], a fact that reflects the etiological relevancy of all clinical entities along the psychosis continuum [14].

**Fig. (1):**
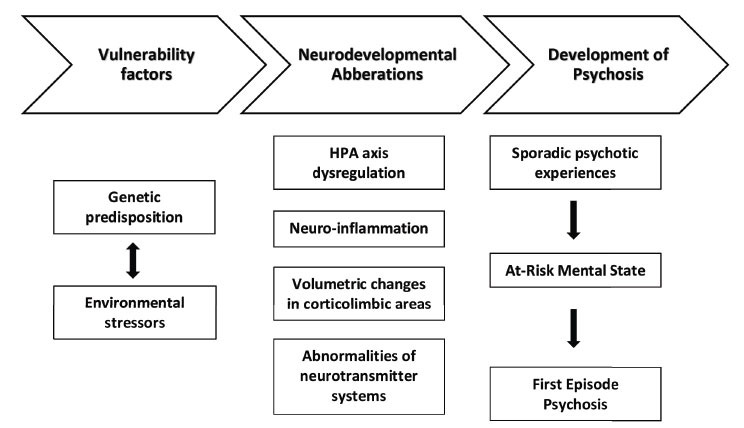
Stress-diathesis conceptualization of psychosis development.

The precise mechanism underlying the gene-environment interaction in psychosis is still elusive. It is suggested that a significant proportion of the disease liability may be due to the impact of the environment upon genetic vulnerability or through gene expression *via* epigenetic mechanisms [15, 16]. The role of stress, as it originates from hazardous environment contexts or factors, is well documented for psychosis onset [17, 18]. However, the exact biological mechanisms by which stress can affect brain function and biological pathways related to psychosis are not fully understood. Early exposure to stress and trauma of genetically susceptible individuals might influence the critical period of brain development and modify key neural and inter-neural pathways over different timepoints (prenatal life, perinatal life, adolescence, and adulthood) [19], pushing towards an emotional and psychotic reactivity to future life stress (stress sensitization) [20, 21]. Thus, current theoretical approaches try synthetically to reflect on the causal complexity of the disorder through biopsychosocial models, highlighting that the type, timing, and severity of stress have a potential role in psychosis development [9]. The diathesis-stress conceptualization summarizes the hypothesized chain of events as follows: exposure of a genetically vulnerable population to adverse environmental conditions has a deleterious impact on stress regulation, resulting in hypothalamic-pituitary-adrenal (HPA) axis abnormalities and peripheral inflammation that further disrupt dopaminergic and glutamatergic pathways, causing misattribution of salience to stimuli implicated in the emergence of psychosis (Fig. **1**) [22-24]. Here, we aim to comprehensively review the literature evidence regarding the relationship between stress, environment, and early psychosis.

## 2 BIOLOGICAL BACKGROUND OF STRESS AND PSYCHOSIS

### 2.1 Stress and HPA Dysregulation

Stress is a fundamentally instinctive adaptive mechanism to an internal or external threat to an organism’s homeostasis, and the neurobiological cascade shares a protective role, but it may, on the other hand, negatively impact brain health in case of dysregulation of related neurobiological systems. The HPA axis and its glucocorticoid end products, *i.e*., cortisol and corticosterone, consist of the main biological system involved in the coordination and synchronization of daytime and sleep-related regulation of the organism’s response to stress and facilitate adaptation [25]. In psychosis, evidence suggests that early-life stress exposures result in aberrant neurodevelopment of brain circuities, including key components of the HPA axis stress response system, such as the amygdala, hippocampus, and medial prefrontal cortex, affecting emotion and regulation of response to stress [26]. Exposure to early adversities, *i.e*., childhood abuse, has been associated with a blunted cortisol awakening response and less reactive HPA [27]. Cognitive deficits, especially in domains of verbal memory and processing speed, have been reported in first-episode psychosis (FEP) patients with low cortisol awakening response [28], and this has been attributed to possible deleterious effects of stress and glucocorticoid on the brain, particularly at the level of the hippocampus [29]. Neuroimaging studies in FEP patients indicate that aberrant cortisol concentration could affect brain structure by causing a reduction in the thickness of the posterior cingulate and a greater hippocampal volume increase over time [30]. Reduced HPA axis responsivity has been correlated with smaller grey matter volumes in the frontal, parietal and temporal cortex as well as in the hippocampus and amygdala in both high-risk and affected individuals [23, 31-33]. Pituitary volume enlargements, as a marker of HPA axis dysregulation, have been reported across all stages of psychosis [34] and in high-risk individuals, and are more pronounced among those who later develop psychosis [35]. Apart from the volumetric changes reported in the limbic areas that regulate the central part of the HPA axis (PFC, hippocampus, and amygdala), exposure to environmental stressors early in life resulting in aberrations in cortisol secretion and daily stress sensitivity has been linked to alterations in brain connectivity [36]. Research work has found a significant association between childhood maltreatment and lower resting state functional connectivity (rs-FC) between the hippocampus and amygdala with subgenual cingulate, with negative implications regarding internalizing symptomatology (*e.g*., anxiety/ depression, withdrawal, and loneliness) [19, 37]. At the molecular level, it has been reported that glucocorticoid receptors (GR) located in the HPA axis regulating limbic regions are downregulated in response to stress, possibly as part of a negative feedback mechanism against chronic secretion of cortisol [32]. This reduced sensitivity of target brain cells to glucocorticoids is enhanced by reduced expression of GR, potentially caused by stress-related cortical dysregulation of specific genes involved in the glucocorticoid signaling pathway [38]. Hence, it is proposed that the reduced GR function underlies the excessive and prolonged stress response [32].

Overall, there is evidence that early psychosis patients exhibit HPA axis abnormalities characterized by increased diurnal cortisol, blunted cortisol awakening response, and decreased cortisol response to psychosocial stressors [39]. However, it should be noted that studies on HPA axis activity in psychosis are limited due to evidence of publication bias and characterized by inconsistent findings. HPA axis dysregulation has been associated with increased basal cortisol levels under resting conditions, a reduced response to acute stressors, and widespread disruptions in neurotransmission [40]. A meta-analysis has found increased morning cortisol levels in patients with schizophrenia, but not in FEP patients [41], while other meta-analytic data indicate blunted cortisol awakening response in patients with schizophrenia and FEP [42], but not in high-risk individuals [43]. A review across all stages of psychosis has reported that, despite methodological variations, there is moderate evidence of an association between stress-induced cortisol-blunting response and psychosis, with the strongest evidence observed for those with chronic schizophrenia [44]. Similarly, a meta-analysis of blood and salivary cortisol levels in early psychosis patients found elevated blood cortisol levels and attenuated cortisol awakening response in FEP but not in high-risk individuals [45]. Moreover, HPA axis dysregulation is implicated in many physical and psychiatric disorders [46] that can be triggered or exacerbated by stress, and related neuroimaging alterations are not disease-specific; thus, HPA axis abnormalities cannot be characterized as a specific risk factor for psychosis [47]. The “neural diathesis-stress model” suggests that the HPA axis dysregulation does not act etiologically by elevating psychosis risk but as a mechanistic link by triggering a cascade of neurobiological events resulting in neural circuit dysfunction, including alterations in the inflammatory response and dopamine signaling [18]. Further elucidating stress and HPA axis role in psychosis includes other research domains, such as inflammation [48], oxidative stress [49], and the gut microbiota [50].

### 2.2 Stress and Inflammation, Oxidative Stress, and Gut Microbiota

Over the last decade, compelling evidence suggests an important link between psychosis and immune system dysfunction, suggesting a mediating role of aberrant immunological processes in genetic and environmental risk for schizophrenia. Of note, inflammation in the context of psychosis is studied as a systemic, chronic, low-grade, and undefined origin phenomenon opposed to the transient nature of inflammatory responses to infections or injuries [51]. Similarities in cytokine alterations are found in schizophrenia, but also bipolar disorder and major depressive disorder during all phases of illness, raising the possibility of common underlying pathways for immune dysfunction [52]. In psychosis, the vast amount of evidence indicates that immune dysregulation of the innate immune response (cytokines and microglia) and components of the adaptive immune response (lymphocyte subsets and anti-neuronal cell surface antibodies) compromise normal neurotransmission and may induce aberrant neurodevelopmental and neurodegenerative processes, which can lead to pathological changes in mood, cognition, and behavior [48]. Moreover, since changes in the levels of individual cytokines have been observed following antipsychotic treatment in acute psychotic exacerbation and FEP, the use of medication has to be considered a potential confounding factor when addressing inflammatory markers in early psychosis [53, 54]. Indeed, while immune dysfunction is evident across all stages of psychosis-spectrum disorders [54], an exacerbated inflammatory cascade characterized by elevated proinflammatory cytokine production has been found as independent of antipsychotic medication and present in both first episode (IL-12, IFN-γ, TNF-α, and sIL-2R) and acutely relapsed phase (IL-1β, IL-6, and TGF-β) of psychotic patients [55], while a recent meta-analysis has reported that proinflammatory cytokines (mainly IFN-γ, IL-6 and IL-12) are significantly elevated in drug naïve FEP populations and associated with presenting negative symptomatology [56]. It is argued that brain morphology is differentially impacted by peripheral cytokines in early psychosis [57], and is further associated with cognitive deficits and symptomatology [58]. Given that genome-wide association studies have demonstrated a significant role of major histocompatibility complex genes in schizophrenia, among other genes [59], gene-expression studies of pro-inflammatory cytokines in brain regions affected in psychosis could provide insight into neuroinflammatory mechanisms that could precipitate neuronal degeneration and decrease neurogenesis, leading to morphological changes increasing liability to psychosis onset [60]. The evidence so far does not support the utility of inflammatory markers in predicting transition to psychosis in high-risk individuals [61] or predicting optimal therapeutic outcomes, as the role of anti-inflammatory agents in the treatment of psychosis remains unclear [54]. Nevertheless, elevated levels of IL-6 are present years prior to the onset of psychosis [62] and have been associated with childhood adversities [63]. It has been postulated that inflammation-mediated pathways may be a common pathway for environmental risk factors, both pre-perinatal exposures, such as maternal infection/ depression [64, 65], and childhood/adult adverse environment, such as childhood trauma, cannabis use, and social exclusion [66], increasing psychosis liability. In line with the above, a two-hit model has been proposed for the role of microglia in the primary immune cells in the central nervous system, and according to this, perinatal stress leads to a primed state of microglia activation, and subsequently later, environmental stress triggers pathological overactivation, leading to excessive synaptic pruning, loss of cortical gray matter, and the emergence of psychotic symptomatology [67], even though the evidence of microglia activation related to brain changes, especially in early stages of psychosis, has been questioned in other research papers [68]. Notably, early programming of the innate immune response as shaped during neurodevelopment may provide insight into broader transdiagnostic importance, and explain the comorbidity between schizophrenia and other major psychiatric and somatic comorbidities, such as coronary heart disease and type 2 diabetes [54].

Disrupted inflammation cascade in psychosis is accompanied by increased oxidative and decreased anti-oxidant cellular stress. It has been proposed that insufficient antioxidant defense leads to higher levels of reactive oxygen species produced by mitochondria dysfunction, causing oxidation of cell macromolecules and, thus, leading to altered cell behavior and brain abnormalities found in patients with psychosis [69]. In detail, increased pro-oxidative status, including impaired antioxidant defense, abnormal serum, plasma, and red blood cell (RBC) oxidative stress parameters, development of redox dysregulation, impaired docosahexaenoic acid levels, and impaired glutathione synthesis have been reported in meta-analytic studies across stages and the broad diagnostic spectrum of psychosis with evidence of independency concerning antipsychotic medication. Moreover, some parameters (total antioxidant status, RBC catalase, and plasma nitrite) are explored as state markers for acute psychosis, while others (RBC superoxide dismutase) as trait markers [70]. In FEP patients, total antioxidant status has been found to be significantly lower than in controls [49], and oxidative markers have been associated with poor clinical, cognitive, and neurobiological outcomes in early-onset FEP patients [71]. The importance of anti-oxidant defense in psychosis onset has been emphasized in a recent systematic review, reporting decreased level of non-enzymatic antioxidants, increased level of lipid peroxides and nitric oxides, and a homeostatic imbalance of purine catabolism, while total decreased antioxidant capacity was significantly associated with cognitive deficits and negative symptoms [72]. In early-onset psychosis, low baseline levels of antioxidants, such as glutathione, have been linked with longitudinal decreases in gray matter volume and cognitive deficits [73], while in young patients with reported childhood trauma, high peripheral oxidation status has been associated with smaller hippocampal volumes and more severe symptom profile than those with a lower oxidation status [74]. Notably, research on the beneficial effects of dietary antioxidants, such as vitamins and Omega-3 PUFAs, has provided mixed results without any proven superiority *versus* placebo in terms of favorable outcomes in high-risk or recent onset psychosis individuals [75]. Interestingly, the brain-derived neurotrophic factor (BDNF), which regulates the survival and growth of neurons and influences synaptic efficiency and plasticity, seems also to be involved, since peripheral (blood) BDNF levels have been consistently found to be reduced in medicated and drug-naïve patients with schizophrenia [76], and in FEP patients [77]. Nevertheless, small studies with mixed findings of BDNF levels are reported concerning the high-risk state [78, 79], however, its utility for predicting conversion to psychosis or treatment response is yet to be clarified. The studies suggest that genetic polymorphisms of BDNF genes might interact with early life environmental stressors influencing clinical severity, cognitive deficits, and brain structural abnormalities in psychosis spectrum disorders [80]. Overall, it is increasingly evident from clinical studies analyzing patients’ blood and cerebrospinal fluid samples, neuroimaging, and post-mortem brain tissue that aberrant oxidative balance and inflammation processes interact during critical neurodevelopmental periods, shaping brain vulnerability towards psychosis emergence through altered neuromaturation and abnormal aging processes [81]. A diagrammatic representation of oxidation disruption, inflammatory alterations, and resulting neuroanatomical changes is depicted in Fig. (**2**).

An emerging key player in understanding how stress during critical neurodevelopmental periods increases psychosis vulnerability by shaping contemporary biological conditions associated with low-grade inflammation and oxidative stress is gut dysbiosis. The existing literature indicates that gut microbiota is a complex and individualized ecosystem of microbiomes directly linked with mental health [82]. Although an association between enteropathies and psychiatric disorders has long been recognized, it has been suggested that gut microbes represent direct mediators of psychopathology *via* bidirectional communication paths between the central nervous system and the gastrointestinal tract [83]. As the microbiome is developed alongside brain development from pregnancy until the first few years after birth, it has been postulated that an imbalance in the gut microbiome during the critical neurodevelopmental phase can influence brain maturation processes, including the establishment of the blood-brain barrier, neurogenesis, maturation of microglia and myelination, by triggering aberrant immune activation and systemic inflammation, thereby leading to symptoms associated with major neuropsychiatric disorders [84]. In later life, the balance between the human microbiome and the development of psychopathology is influenced by several factors, such as diet, exposure to medication, sleep disorders, and external stressors [85]. Environmental stressors, such as adulthood trauma [86] and cannabis exposure [87], have been reported to increase psychosis liability mediated by microbiome alterations. Furthermore, there are consistent findings of altered synthesis and function of the microbiome in chronic schizophrenia [88], first-episode, drug-naïve patients [89], and ultra-high-risk individuals [90], as compared to controls. Diversity in microbiota composition in psychosis has been correlated with altered brain structure and function [91], cognitive dysfunction [92], and symptom severity [93]. It should be noted that current data are insufficient to conclude whether microbiome changes can establish the direction of causality associated with increased risk of psychosis or are simply the result of non-specific factors or treatment/ disease-related factors [94], while the potential therapeutic role of dietary supplements is yet undetermined [95].

### 2.3 Stress and Psychosis Onset at the Level of Brain Signaling

The leading biological model of schizophrenia is the dopamine hypothesis. This theoretical framework suggests that enhanced striatal dopamine synthesis capacity and stress-related increased dopamine release are predominantly accountable for the neurochemical abnormalities that mediate psychotic symptoms [96]. Another influential theory regarding schizophrenia onset is the glutamate hypothesis, where it is proposed that acute and chronic stress, specifically the stress-induced release of glucocorticoids, causes changes in glutamate neurotransmission in the prefrontal cortex and the hippocampus, possibly through hypofunction of the NMDA receptors, with psychosis-related implications [40]. These etiopathological configurations seem to complementary encapsulate the different symptomatic dimensions of psychosis, since increased dopamine synthesis and release have been firmly associated with positive symptoms, while abnormalities in glutamatergic neurotransmission have been connected with negative symptoms and cognitive dysfunction [23, 33]. In accordance with this, the diathesis-stress conceptualization posits that environmental stressors activating the HPA axis trigger a cascade of events resulting in neural circuit dysfunction that affects dopaminergic and glutamatergic reactivity, making individuals more prone to develop psychosis [33]. Indeed, research work has pointed out a significant association of stress sensitization due to environmental adversities, such as childhood abuse, neglect and migration with elevation in striatal dopamine synthesis [1], and acute stress as well as cannabis use, with abnormal glutamatergic neurotransmission [97].

Another neural signaling worth addressing is the endocannabinoid (eCBs) system, as the increased incidence of cannabis-induced psychosis has pointed out the relevance of this neurochemical pathway with schizophrenia. Indeed, it has been found that enhanced cannabinoid CB1 receptor density in corticolimbic areas, along with abnormalities in the levels of endogenous ligands of CB1 receptors, such as anandamide, might be interconnected with other neurochemical pathways linked to psychosis [1, 98]. In addition, endocannabinoid signaling involved in stress-sensitive nuclei of limbic structures (hypothalamus and amygdala) possibly reflects the substantial role of eCBs in the regulation of stress responses [99]. Taken together, these findings allow us to revisit the neurochemical models of schizophrenia by integrating the “cannabinoid hypothesis” into the classic “dopaminergic and glutamatergic hypotheses” [1]. It has to be noted that the dysregulation of this neurochemical pathway has an endogenous and an exogenous aspect. That is, regardless of cannabis use, dysregulation of eCBs endogenously, due to genetic and/or early life exposure to cannabinoid agonists or fear-evoking stimuli, might be a risk factor per se, pushing towards psychosis; on the other hand, cannabis use represents an exogenous risk factor, which by impacting on the reactivity of the eCB and the other psychosis-related neurotransmitter systems (dopaminergic and glutamatergic), might cause neurobiological effects favoring psychotic phenomena [98, 100, 101].

## 3 ENVIRONMENTAL RISK FACTORS

Exposure to environmental risk factors can occur at a number of points over time, acting on an individual or on a population level and representing either a direct determinant or an indicator of the increase in the likelihood of developing primary psychosis. These risk factors can interfere with neurodevelopment or display a stressful traumatic impact that overwhelms the emotional coping mechanism of the susceptible individual. Since the exact pathophysiology of psychosis is still unspecified, we consider risk factors as correlational and not causal [102]. In the current review, we have addressed environmental adversities that might take place from time of conception to onset of illness, following the temporal classification of early life, childhood and later life (Table **1**). It must be noted that several risk factors could act at various points throughout the premorbid and prodromal period of the disease [103]. In addition, some risk factors, such as disadvantaged social conditions or low educational level, might precipitate the occurrence of other risk factors, such as a higher number of obstetric complications, more stressful life events, infections, poor maternal health conditions, and poor medical monitoring [104].

### 3.1 Early Life Environmental Stressors

#### 3.1.1 Parental and Sociodemographic Factors

Several socio-demographic factors have been identified to increase the likelihood of psychosis. Parental age at the time of birth has been associated with psychosis [1, 105]. Maternal age has been both positively (<19 years and >35 years) [106-108] as well as negatively (>30 years) [109] linked to increased risk. On the other hand, advancing paternal age has more consistently been positively associated with psychotic experiences [108, 110-112], with the risk rising particularly after > 45 years of age [4]. It has been hypothesized that the accumulation of sporadic *de novo* mutations in paternal germ cells over time might be responsible [113]. Alternative explanations implicate schizotypal personality attributes of late fatherhood [114] that might be transmitted to the offspring as heritable traits favoring the emergence of psychosis [115].

Another risk factor for psychosis that has been repeatedly suggested is birth in winter and early spring in the Northern hemisphere [116], although the association seems to be small [4, 117]. The mechanisms involved are hypothesized to be secondary to other adversities (*e.g*., obstetric complications, variations in light, low temperature, nutritional deficiency, maternal infections), but with no specific findings so far [1, 103]. Other sociodemographic factors that have been discerned are urban birth and being born in a socioeconomically disadvantaged environment. Again, the mechanisms involved are hypothesized to be the result of in-utero exposure to environmental stressors (*e.g*., less access to medical resources, including suboptimal prenatal care, maternal stress with potential effects on the fetus, early exposures to toxins, infections, or nutritional difficulties) [105, 108, 118-120].

#### 3.1.2 Perinatal Factors

Pre-natal and peri-natal risk factors have been extensively studied and repeatedly connected to psychotic experiences [102, 104, 105, 121]. The relationship between obstetric complications and psychosis seems to be enhanced in individuals with an early onset of illness [103, 122]. These early life stressors have commonly been divided into a) pregnancy-related, including maternal health problems and abnormal fetal growth, b) delivery-associated, and c) perinatal. The pathophysiological mechanisms that have been proposed involve hypoxia, fetal malnutrition, gestational infections, and premature birth [1]. Activation of the immune system through oxidative stress and neuroinflammation could mediate the effects of psychotic risk [105]. Moreover, stress biomarkers, such as dysregulated cortisol levels and elevated markers of inflammation, including C-reactive protein and interleukin-8, have been found in mothers of offspring with schizophrenia [123-125]. The implications of the pathogenic mechanisms are hypothesized to impact neurodevelopmental processes, including neurogenesis, proliferation, and migration of neural cells, synaptogenesis, gliogenesis, and myelination of subcortical areas, possibly constituting the first-wave hits for the emergence and progression of psychosis [102]. Indeed, several studies have demonstrated an association between the presence of structural brain abnormalities on imaging and history of obstetric complications in samples of subjects with schizophrenia, although these observations have not always been confirmed [103]. In addition, another study suggested that the interactions between genes participating in the cellular stress response are selectively expressed in the placenta and the exposure of specific intra-uterine complications may increase the risk of developing primary psychosis [121]. This prenatal gene-environment interaction might adversely affect placental transcriptome and, consequently, placental functioning and fetal development, with male fetuses presenting higher vulnerability [121]. However, Vassos *et al.* [126] found no support regarding the potential mediating effect of placenta biology in the genetic track of schizophrenia.

Regarding maternal physical health, gestational insults, such as bleeding, preeclampsia, rhesus compatibility [9, 103, 127], diabetes or obesity during pregnancy [4], maternal anemia [128], and maternal nutritional deficits either involving micronutrient intake or food deprivation [103, 108], have been implicated [105]. Other pregnancy-related adversities that have been found to be associated with psychosis onset are maternal stress [108, 129], smoking, and rhesus incompatibility [103]. Stressful events during pregnancy have particularly been indicated as consistent risk factors for psychosis in offspring; however, the results have not been replicated, indicating that considerable caution must be exercised in drawing conclusions on the role of maternal stress during pregnancy [103]. With regards to prenatal and perinatal infections, viral and other infectious agents, such as influenza (especially in the first trimester of pregnancy) [123], rubella, toxoplasma gondii [130], HSV type 2 [131], and other unspecified maternal infections [105] contracted during pregnancy [132, 133] and around the time of conception [134] have also been pointed out to affect psychosis onset in offspring [104], although the findings have not entirely been consistent as shown in review and meta-analysis studies [103, 135]. Other obstetric complications that have been reported to be associated with risk for psychosis are hypoxic-ischemic events, polyhydramnios [108], uterine atony, preterm rupture of membranes, emergency cesarean section, asphyxia [4, 9, 108, 127], and use of forceps [122].

### 3.2 Childhood Trauma

Childhood trauma (CT) is considered a deeply distressing event causing actual or potential harm to a child due to accountability or omission of the child’s caregiver [136]. The incidence of adverse events causing traumatic experiences could affect more than one-third of the general population [125, 137]. In recent years, the impact of CT has been broadly investigated with respect to the risk of mental health disorders. Trauma models of psychiatric diseases have pointed out the effect of different types of traumatic stress in early life and childhood as a key risk factor [105, 138]. Regarding psychosis, the role of CT as a potential contributor to psychosis risk has been highlighted, with numerous studies confirming the association [139-145]. It seems that individuals with psychotic experiences are significantly more exposed than the general population. Several studies have shown that patients with psychosis are three times more likely to report childhood trauma in comparison to healthy controls [6, 146]. In addition, a study involving subjects with schizophrenia showed that 86% of the patients reported at least one childhood adversity [147]. History of CT has also been associated with both healthy people experiencing psychotic-like phenomena and ARMS subjects [102]. In fact, CT has been found to predict the transition from ARMS to frank psychosis [139] and to increase the severity of positive psychotic symptoms [148]. It is advocated that the potential removal of CT would result in >30%-reduction in the number of psychosis cases [6].

CT associated with psychosis risk includes abuse (physical, sexual, and emotional), neglect (physical or psychological) [4, 14, 128, 149, 150], active and/or passive peer victimization (in particular bullying) [14, 149, 151], and exposure to the familial disrupted environment, including parental separation or death [6, 14, 149], incarcerated family member, the witness of domestic violence [125], developmental challenges associated with foster care [152], and parental communication deviance that has been confirmed as a risk factor irrespective of familial genetic susceptibility [105]. Traumatic brain injury has also been considered as a possible risk factor. It has been postulated that childhood head injury can play a facilitating role in the onset of primary psychosis [4, 128]. A retrospective study showed subjects with schizophrenia to have higher rates of childhood history trauma compared to their siblings [153]; still, reports are not consistent [154, 155]. The proposed explanation is either a direct impact that causes anatomical alterations in the CNS, favoring the onset of the disorder, or that ARMS subjects might be more susceptible to accidents [105, 156].

Despite the strong evidence linking CT to psychosis onset and severity, the conceptual framework defining the relationship is not strictly defined. Considering the diathesis-stress model of psychosis, a body of literature suggests that the biological sequelae of childhood abuse are reflected in stress sensitization through aberrant HPA axis activation, predisposing susceptible individuals to a disproportionate reactivity towards later life stressors [16, 157]. The second-wave hits of traumatic adversities occurring during childhood and adolescence coupled with high-perceived stress may result in affective dysregulation [102]. Indeed, the relevance of the affective pathway between trauma and psychosis has been extensively discussed [16, 158]. Emotion regulation is based developmentally on a safe, emotionally attuned, and regulated caregiving milieu. CT, particularly of a familial interpersonal nature, impedes the cultivation of adaptive emotion regulation strategies, leading to emotional dysregulation [148, 158]. This affective dysregulation has been associated with reality distortion [159] and projection of negative meaning to neutral stimuli, resulting in disproportional reactivity to minor daily stressors and maladaptive evaluation of external reality in susceptible vulnerable individuals [16, 160, 162].

It must be noted that CT and adversity rates are significantly higher for children living in urban areas and/or growing up in socioeconomically disadvantaged groups [1, 125]. Moreover, the increased risk of psychosis due to exposure to CT seems to follow a dose-response pattern. Specifically, research has highlighted a possible compounding effect, showing that the association with psychosis risk is enhanced with the increase in the number and type of adverse experiences [163]. That is, the frequency of childhood environmental stressors paves the way for further traumatization or revictimization and is related to both the frequency and severity of psychotic phenomena [21, 149, 163, 164]. In addition, research work following the interaction of CT with other psychosocial stressors underlines the mediating role of social defeat in the association of childhood adversities and psychosis [165] or the mediating role of CT in the association of sexual minority status and psychosis risk [165, 166]. Still, other studies suggest that the coexistence of urbanicity, cannabis use, and childhood trauma has an additive impact on the risk for the emergence of psychosis [167]. Additionally, the impact of CT on psychosis risk is affected not only by the interaction between different types of trauma, but also by factors such as age, frequency, and duration of exposure to the traumas. Consequently, the dose-response effects of trauma have to be considered as explanatory factors related to the escalation of psychosis risk [105]. Regarding gender differences, there are mixed results with reference to the impact of CT on psychosis onset. Females have been reported to be more prone to develop psychotic phenomena after childhood adverse experiences, especially sexual abuse, while other findings highlight the male vulnerability to the potential psychotic consequences of childhood adversities [12, 148]. It is possible that gender differences in the psychological processing of traumatic events, such as internalization of emotional burden with dissociative response *versus* hyperarousal and externalization with the fight-flight response, might influence the phenomenological output of psychotic phenomena [16, 168]. However, no straightforward conclusions can be drawn.

### 3.3 Late Environmental Factors

As the stress-vulnerability theory posits, the already premorbidly-shaped sensitivity provides a biological substrate for further stress-related environmental insults that may precipitate or bring forward psychotic symptomatology. Consistent with the diathesis-stress model, the two-hit hypothesis (*i.e*., early genetic and environmental discrepancies produce long-term vulnerability to a “second stress hit” that leads to the emergence of symptomatology) and its meta-evolved three-hit hypothesis (*i.e*., hit-1: genetic predisposition, hit-2: early-life environment, and hit-3: later-life environment) suggest that, in a given environmental context, vulnerability is enhanced and symptomatology emerges when failure to cope with adversity accumulates [169, 170]. Genetic predisposition and early-life adversity affect normal neurodevelopment [171, 172] and may be predictive of later hazardous exposures [173, 174] and the development of maladaptive coping mechanisms [175]. In this context, late psychosocial stressors, accumulatively and repeatedly, lead to further dysregulated stress response and sensitization of key neurobiological pathways to psychosis [22].

The chronological differentiation amongst early and late environmental stressors is vague, given that known psychosocial risk factors, such as trauma, urbanization, cannabis use, and migration, could imply a burdensome role in any neurodevelopmental stage during brain maturation prior to psychosis onset. Nevertheless, it is plausible to argue that early life risk factors are more consistent with the neurodevelopmental model of schizophrenia, while later environmental risk factors are explored not only as potential etiological factors but also as both precipitants of illness and modifiers of the course after illness onset.

#### 3.3.1 Social Disadvantage and Social Defeat

The sense of belonging and being part of a broader social system is fundamental for mental resilience [176]. While social adversities could occur in different forms (unemployment, poverty, low social class, minority status, poor access to health care), both the direct effects of adversity and the individual perception of social disadvantage/isolation are implicated in psychosis risk [103]. Studies report high indirect markers of social disadvantage/isolation in FEP patients, such as being single or unemployed, living alone, in rented accommodation, in overcrowded conditions, and receiving an income below official poverty, not only at first contact with psychiatric services but up to 5 years prior to the onset of psychosis, with around a twofold increased odds [177]. Low socioeconomic status has been linked to increased psychosis risk, and it is indicated that personal rather than parental socioeconomic disadvantage implies the greatest impact on the onset of psychosis [178], however, literature evidence has not been conclusive to support this association [179]. Another source of social disadvantage is low perceived social capital, which is defined as each individual’s immediate neighborhood participation and is further described by dimensions, such as civic disorder, the impact of civic disorder, informal social control, and social cohesion and trust [180]. Low levels of social capital have been related to subsequent psychosis risk, both as a stressful condition per se [181] and as exacerbating the effects of other psychosocial factors [182].

The effect of social disadvantage, exclusion and isolation, as a perceived negative experience, on psychosis risk has led to the formulation of the social defeat hypothesis [183, 184]. Researchers argue that the “negative experience of being excluded from the majority group” provides a parsimonious and plausible explanation for a number of environmental psychosis risk factors [185], including migration, low intelligence, illicit drug use, urban upbringing, childhood trauma, but also other factors as African-American ethnicity, unemployment, single status, hearing impairment, autism, illiteracy, short stature, Klinefelter syndrome, and possibly, sexual minority status [184]. It is argued that the crucial determinant of social exclusion leads to sensitization and/or increased baseline activity of the mesolimbic dopamine system and, thereby, to an increased risk of psychosis, and this has been further confirmed by animal studies [186].

#### 3.3.2 Urbanicity

Urbanicity has been suggested as among the significant contributors to the development of psychotic disorders [4]. The multidimensional construct of urbanicity concerns social and economic stressors (*e.g*., migration, ethnic density, income/employment/education inequality, social fragmentation), nature exposures (infections, exposure to pollutants, diet, lack of green space), and access to hazardous resources (illicit drug use) [187]. Living in an urban environment has been found to increase the risk of developing psychosis approximately 2.37 times more compared to living in rural settings; furthermore, the population density has been associated with higher psychosis risk in a dose-response manner, suggesting possible causality [188]. During childhood, factors, such as residence changing from rural to the urban environment [189] and the duration of years spent in big cities, are reported to increase the risk of developing psychosis [190]. It is argued that the urbanization-psychosis association is not just an epiphenomenon of the social drift of patients with psychosis who migrate to big cities, but other confounders are also involved. Well-designed studies have reported elevated rates of psychotic disorders in densely populated areas to control for the confounding effect of other psychosis risk factors, such as substance abuse or ethnic minority status [4, 188].

Recent review studies indicate that urbanicity-psychosis associations are heterogeneous and driven by multiple risks and protective factors that seem to act differently in different ethnic groups and countries [187]. Robustness of the urbanicity-psychosis association has not been replicated in epidemiological studies comparing developing countries with low- and middle-income countries [191]. The epistemological debate about the conceptual validity of urbanicity, the mechanism of causation, and the problem of residual confounding remains open [192]. A review by Vargas *et al.* provides a broader systems-level perspective suggesting three different types of exposures: stimulation exposures, including urban environments with high population density and crime exposure; deprivation exposures, including environments lacking socioeconomic, educational, or material resources; and discrepancy exposures, including ethnic density, income inequality and social fragmentation [24]. Different types of exposures are suggested to impact differently on biological pathways related to psychosis, *i.e*., stimulation exposures characterized by heightened chronic physical arousal and vigilance could evoke feelings of threat and symptoms of paranoia affecting the structure, function, and connectivity of threat circuit regions, including the amygdala, hippocampus, and ventromedial prefrontal cortex; deprivation exposures *via* mechanisms lacking important environmental enrichment stimuli that enhance adaptive neurodevelopment may be related to over-pruning and protracted prefrontal cortex development; discrepancy exposures may provoke feelings of social exclusion and lack of empathy and could impact the oxytocinergic system [24].

#### 3.3.3 Migration and Ethnical Minority

Migration has been reported as one of the environmental hazards near the onset of illness, with an observed increased incidence of psychosis among some migrant groups compared to psychosis patients with no family or individual history of migration [193]. First- and second-generation migrants from countries outside Europe have approximately three times higher risk for developing non-affective psychosis than that for the local European population [194], and this risk appears to persist into the second generation [195]. Specific ethnic groups are of higher risk, and meta-analytic evidence suggests that black Caribbean ethnic groups in England have a 4.7-incidence rate ratio of developing psychosis relative to the baseline population in England [196]. Of note, there is no evidence that reported risk could be explained by an increased incidence of psychosis in countries of origin, but the magnitude of this risk increase varies by minority group and by host country [195]. Interestingly, an older study has shown that the risk of developing the disorder is much lower in siblings of Afro-Caribbean migrants with schizophrenia living in the Caribbean than in those who reside in England [197], indicating that environmental factors in the European host country, but not in the country of origin, should be of higher importance. Moreover, Afro-Caribbean ethnicity has not been related to an increased risk of psychosis in countries other than England [198], indicating that genetic predisposition cannot explain the findings, but specific environmental factors related to the host country are more influential.

The majority of studies indicate that environmental factors mainly associated with the post-migration phase increase the risk of psychosis among migrants. Amongst them, low ethnic density, discrimination, social exclusion and disadvantage, linguistic distance, and lack of access to educational and occupational opportunities are reported as ethnical-related adversities [1, 199]. The risk for psychosis increases not only for non-white minorities with the decrease in their proportion in the local population due to lack of social support and high exposure to discrimination reported as important mediators for this finding [200], but also for other ethnic groups when they comprise a small proportion of the neighborhood’s population [201]. Distinctive social contexts for specific minority groups in specific host countries may explain heterogeneity in effect sizes for the risk of psychosis amongst ethnic populations [202]. From a neurobiological perspective, elevated striatal dopamine function, increased inflammation, and increased cortisol levels have been associated with migration and ethnical minority status [43, 66, 203, 204]. The sociodevelopmental hypothesis posits that genetically predisposed individuals from certain migration/ethnic groups are further pushed to psychosis through mechanisms of dysregulated dopaminergic neurotransmission and increased stress sensitivity, manifested with social cognitive biases, paranoid ideation, and other psychosis-like experiences [205].

#### 3.3.4 Cannabis Abuse

Amongst drugs associated with elevated psychosis risk, such as cocaine and amphetamines [206], cannabis has been the most well-studied and robustly linked with both etiology and the precipitation of psychosis onset [14]. Especially high-potency cannabis users have an estimated threefold increased risk [207], while a dose-response association between level of use and psychosis risk has been reported [208]. Moreover, differences in both frequency and quality of cannabis use may partially explain the variation in the incidence of psychotic disorder across different geographical regions [209]. At an individual level, cannabis use has been linked with earlier psychosis onset by about 3 years compared to non-users [210] and compared to affective psychosis patients [211], or even earlier with 6 years average when the severity of use (daily use, high-potency cannabis, synthetic cannabinoids) is considered [212], while age of cannabis exposure may be predictive of the age of later psychosis manifestation [213].

Even though the association between cannabis use and psychosis onset is well documented, the underlying biological mechanisms are yet to be clarified [214]. It remains unclear whether psychotic predisposition leads to higher cannabis use for self-medication purposes or whether cannabis itself induces psychosis proneness. While a shared, common, genetic liability for both schizophrenia and cannabis use cannot be excluded [215], it is supported that genetic predisposition to schizophrenia explains only a small proportion of cannabis use in the general population or in patients. Evidence for a gene-environment additive effect of cannabis exposure to high polygenic schizophrenia score for the development of psychosis is growing [216], while the argument of self-medication cannabis use amongst psychosis patients has not been confirmed [217]. From a neurobiological perspective, cannabis use is hypothesized to induce postsynaptic dopaminergic supersensitivity in the associative striatum, and this is further reinforced by evidence of genetic studies of genes implicated in dopaminergic neurotransmission that are associated with cannabis-induced psychosis [218]. Functional imaging studies have found an association of cannabis use with reduced inhibitory control by attenuating inferior frontal activation during response inhibition tasks and with modulation of the neural substrate of salience processing, which are both implicated in psychosis symptomatology [214]. Moreover, the impact of exogenous cannabinoids on the endocannabinoid system is explored, and the down-regulation of cannabinoid CB1 receptors after chronic and recent cannabis exposure has been the most well-established finding [219]. It is argued that cannabis use and chronic stress can disrupt the cannabinoid system leading to abnormal stress regulation by suppressing the HPA response to stress, thus leading to psychosis emergence in vulnerable individuals [220].

#### 3.3.5 Life Events

The role of life events in precipitating psychosis has been well identified from older [221, 222] and more recent studies [223, 224]. While there is an inconsistency in their definition in research, life events are investigated as major changes in one’s adult life, which an individual perceives as stressful, threatening, uncontrollable, exceeding usual coping ability, and may include exposure to serious illness or accident, death of a close friend/family member, financial crisis, intrafamilial or relationship conflicts, separation or divorce, occupational crisis, and legal issues [225]. Meta-analytic evidence suggests a threefold increased odds of life events in a period between 3 months and 3.6 years preceding psychosis onset [225]. It has also been supported that an intrusive nature of the life event, *i.e*., physical assault or invasive operation, is more highly correlated with psychosis onset, perhaps leading to a hostile perception of the social environment [225, 226]. Moreover, an increased number of threatening life events has been associated with increased psychosis risk in a dose-response manner 1 year prior to psychosis onset [227].

Stressful life events are universal, with about 40% of the population experiencing a major event each year [228]; however, only a very small amount of the population develops psychosis. The combination of exposure, as a threatening event in an individual’s life, and sensitivity, as an impaired tolerance to normal stress or increased sensitivity to daily stressors [229], may predispose an individual to psychotic symptomatology. It has been argued that psychotic patients have an increased stress sensitivity and lower stress threshold compared to the normal population [230] and compared to patients with other psychiatric disorders [231]. A recent study has emphasized the role of stress sensitivity as a putative risk mechanism linking negative life events and unfavorable mental health outcomes [232]. Moreover, exposure to recent stressful life events has been associated with poorer mental and physical health in individuals with more severe environmental, but not genetic, liability for schizophrenia [233]. Even though the exact neurobiological mechanisms governing the association of life events and psychosis remain elusive, the stress-diathesis model [23, 234] posits that exposure to psychosocial stress, such as recent life events or previously adverse experiences, may cumulatively increase the behavioral and biological response to subsequent exposures, *via* HPA dysregulation, dopamine sensitization in mesolimbic areas, and increased stress-induced striatal dopamine release [18].

## 4 CUMULATIVE EFFECT OF ENVIRONMENTAL STRESSORS

The multidimensional role of environmental stressors in psychosis onset has been recently examined under the general prism of total exposure score. Most literature evidence originates from one-exposure-one-outcome approaches, *i.e*., the association of isolated exposures, such as childhood trauma, with psychosis, which has not provided holistic insight into the total environmental impact on psychosis. Recently, different research approaches have suggested to construct an aggregate index or weighted sum of the total number of risk factors under the rationale of a polygenic risk score for schizophrenia, although its utility has been questioned due to its low predictive power and lack of population diversity [235]. The polyenviromic risk score [236], the Maudsley environmental risk score for psychosis [237], and the exposome score for schizophrenia [238] are among the designed tools approaching the cumulative environmental risk and the interconnected network of nongenetic exposures an individual is exposed to across lifetime. Even though literature is still scarce, the predictive utility of such polyenvironmental risk tools is explored with encouraging findings associated with clinical outcome measures, such as conversion to psychosis [236], diagnosis [239], and clinical outcomes [240].

Another approach expanding the knowledge base regarding causality inferences is mendelian randomization (MR). This method utilizes genetic markers associated with the exposure as instrumental variables to provide causal estimates between exposures and disease outcomes while testing for the presence of pleiotropy (association of genetic variables with the outcome through more than one causative trail) [241]. Findings from MR analyses in the field of early psychosis, particularly in the context of cannabis, and more recent exposome-wide studies have pointed out, on the one hand, forward associations with exposure to sexual abuse and pleiotropy of risk-taking behavior, and on the other hand, reverse associations without pleiotropy of exposure to physical abuse as well as cannabis use and reverse association with pleiotropy of worrying too long after embarrassment [242]. The causal relationship between cannabis use and schizophrenia onset through MR has been replicated [241], and a possible bi-directional association (*i.e*., relation of schizophrenia’s genetic predisposition per se to increased use of cannabis) has been underlined [243]. Nevertheless, the specific association of the individual, total, and aggregated environmental burden with stress-related biological mechanisms is yet undefined.

## 5 PROTECTIVE FACTORS AND RESILIENCE

The main body of literature has focused on risk rather than protective factors, and an inconclusive level of evidence has been reported for candidate protective factors [4]. Protective factors, individual, genetic or environmental, have been defined as “factors that modify, ameliorate or alter a person’s response to some environmental hazard that predisposes to a maladaptive outcome”, and could ameliorate the detrimental effect of risk factors or even protect biologically predisposed individuals [244] from like risk factors acting across all stages of neurodevelopment. Specifically, maternal age between 20-29 years, maternal nulliparity, increased birth weight (> 3500 gr) [108], and adequate dietary supplementation with vitamin D, iron and folates [245] are reported as possible prenatal and perinatal protective factors for psychosis. Healthy communication and bonding between parents and children have been found to reduce the risk of developing psychosis in subjects at high genetic risk for schizophrenia [246]. Positive family environment and support have been reported as predictive of symptom improvement in individuals with high risk for psychosis [247], especially at early stages where important foundations of becoming social are influenced by parental bonding and parenting styles [248]. Partnership, optimism, humor, openness, extraversion, and emotional stability have been found to lead to lower levels of distress in individuals with psychotic experiences [249]. Greater social support, engaging in physical activity, and greater neighborhood social cohesion have been associated with reduced risk of developing psychotic experiences in poly-victimized adolescents [250]. Employment has been reported to reduce the risk of transitioning to psychosis in clinical high-risk individuals [251], and elevated socio-economic status and financial well-being have been associated with a lower psychosis risk [105].

**Fig. (3):**
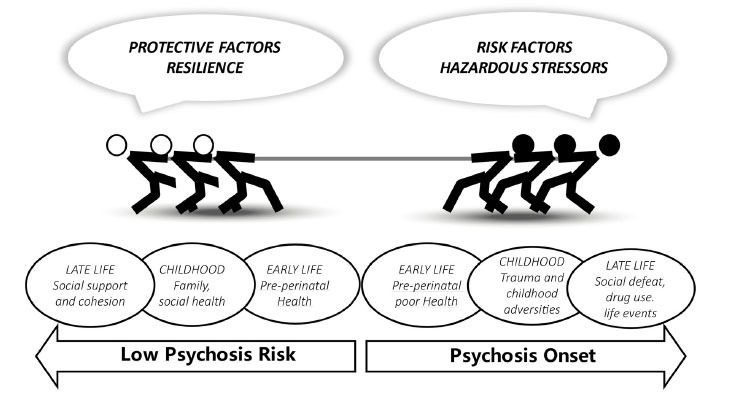
Schematic of the hypothesized impact of risk *vs*. protective environmental factors on psychosis risk across developmental stages.

Psychoenvironmental stressors do not necessarily increase psychosis risk per se, as associated coping and perception is equally important [252, 253]. Stress regulation can be either detrimental or protective and precipitate or ameliorate psychotic phenomena. Coping can be defined as a goal-oriented or intrapsychic effort to face, manage, reduce or tolerate stressful events or a stressful environment [254]. Perceived stress relates to how the same objectively stressful event is dealt with in different ways and is helpful in understanding the individualized experience of predicting and controlling thoughts and feelings under stressful situations [255]. It is indicated that psychosis patients adopt maladaptive strategies in order to cope with symptoms, such as self-blame, avoidance, drug use, and social seclusion, across all stages of the disorder [256, 257], and they are commonly characterized by cognitive bias, such as attributional bias [258], threat anticipation [259], and jumping to conclusions [260]. On the other hand, in order to enhance resilience, implementing positive coping strategies has been associated with lower self-stigma [261], fewer negative symptoms [262], better quality of life [263], fewer depressive symptoms, and better neurocognitive performance [264] at various stages of illness. Social competence, problem-solving, autonomy, and sense of purpose are among major determinants of personal resilience [265], and meaning in life has been suggested as a fundamental protective factor in the context of psychosis-related psychopathology [266]. Moreover, improving daily stress sensitivity and coping with life events by focusing on emotion regulation has been suggested as a resilience-enhancing strategy, especially prior to psychosis onset [232]. This struggle between socioenvironmental risk and protective factors, burdensome stressors, and adaptive coping can be depicted as a tug of war across developmental stages prior to psychosis onset (Fig. **3**).

## 6 BACKGROUND OF THE GENE-ENVIRONMENT INTERACTION MODEL OF PSYCHOSIS

Different conceptual templates have been put forward to describe the pathophysiological framework of psychotic spectrum disorders. The classic neurodevelopmental model posits that psychotic disease is the result of aberrant brain development and maturational processes, leading to alteration of brain structure and function that takes place before the onset of the disease [267, 268]. The diathesis-stress model outlines the interaction between an individual’s inherent vulnerability (*i.e*., diathesis) to develop psychosis and his/her experience of environmental stressors. The severity of environmental stress required to trigger psychotic phenomena depends on the extent of the inherent vulnerability of the individual [234, 269]. Another approach to explain the emergence of psychosis is the stress-sensitization model, according to which genetically susceptible individuals become sensitized to stress early in life through environmental adversities, such that the level of stress needed to trigger psychotic phenomena overtime becomes progressively lower [270]. The experience of stressful life events and early trauma have set the stage for stress sensitivity, in the context of which, later in life, minor daily stressors might trigger psychosis onset [230]. This dysregulated response to stress resulting in heightened emotional reactivity is found to be independent from cognitive impairments in subjects vulnerable to psychosis, suggesting an affective pathway to psychosis [16]. In addition, a “multi-hit” model has been brought forward, outlining the association between exposure to environmental stressors during the particularly sensitive time period of early brain development and maturation during adolescence, precipitating psychotic biological and phenotypical sequelae [271]. All these etiological conceptualizations address cohesively the gene-environment interplay, acknowledging that a sequence of unfavorable events occurring during early (perinatal, childhood) and late (adolescent) development operate on the background of genetic risk for psychosis, be it a result of an underlying variation in DNA sequence or an epigenetic variation in gene expression [225].

Psychosis-related gene-environment interactions involve multiple common genetic variants in multiple genes with very small effect sizes and various environmental factors constituting a dense network of exposures [272]. Earlier research work used candidate gene studies or individuals with either familial history of psychosis spectrum disorders or schizotypal personality disorder as a proxy for elevated genetic risk for psychosis and different environmental stressors to investigate gene-environment interaction. However, the GWAS research approach that clarified the polygenic architecture of the disease allowed for polygenic risk scores for schizophrenia (PRS-SCZ) to be developed, summarizing the level of genetic risk of an individual [273]. Likewise, to better encapsulate the multifaceted environmental exposures, cumulative environmental exposure scores for schizophrenia (ES-SCZ) were developed [274]. The use of aggregate scores as measures of collective genetic and environmental liability further enhanced the investigation regarding the link between genes, stress, and childhood adversity in psychosis development.

Results so far suggest that the gene-environment interplay in psychosis is characterized by a positive dose-dependent additive interaction, indicating synergy between genetic susceptibility and environmental adversities, especially childhood trauma and cannabis use [2, 9, 162, 216, 272]. Furthermore, it is pointed out that for some individuals, the increased liability to develop psychosis exists only in the presence of both genetic and environmental risks but not when either is present alone [162]. In summary, genetic and environmental risk factors are independently and jointly associated with schizophrenia, and there is evidence that the combined influence of genetic liability and environmental exposure is larger than the sum of the individual effects of each [216].

However, there are substantial methodological difficulties when interpreting gene-environment interaction in psychosis that have to be considered. It has been suggested that possible gene-environment correlation, where the genotype of the individual influences the exposure to environmental stressors, might be the determining factor mediating the association [275] rather than an independent interaction. On the other hand, error-prone statistical models [276], limited range of genetic and/or environmental variation [277], and study-design inconsistencies, such as inadequately powered studies, measurement errors, or flaws in sample selection might fail in revealing genetic and environmental interactions leading to unreliable conclusions [278, 279].

## 7 DISCUSSION

The current review comprehensively presents the extended evidence regarding the close relationship between abnormalities in stress regulation, environmental insults, and psychosis onset. In brief, early stress can sensitize genetically vulnerable individuals to future stress, modifying their risk for developing psychotic phenomena. Biological pathways reflecting the anatomical and neurochemical substrate of the stress response (hippocampal and amygdalar brain regions, HPA axis and immune system, dopaminergic and glutamatergic neurotransmission) provide important mechanisms linking environmental risk factors to the development of psychotic symptoms. Prenatal and perinatal period, childhood, and adolescence constitute critical time windows for neural development that will determine a person’s capability to develop adaptive and coping mechanisms towards everyday challenges. Obstetric complications, traumatic experiences, psychosocial stressors, and cannabis use might negatively intervene with brain developmental trajectories and disturb the balance of important stress systems, which, together with recent life events, push the individual over the threshold for the manifestation of psychosis. The deleterious environmental effects seem to act in a dose-response fashion, meaning that there is a cumulative impact of environmental adversities on the psychosis risk in vulnerable individuals.

Early life insults, such as perinatal infections or hypoxic events, implying a direct pathological impact on brain growth in this sensitive developmental stage might offer a more straightforward explanation regarding the psychosis risk. In later life, the nature of exposures and interrelationships regarding psychosocial factors are more obscure. However, advances in the investigation of the neural basis of CT in early psychosis have focused on brain regions and neurochemical substrates implicated in the regulation of the HPA axis and, hence, emotional reactivity, suggesting that a history of CT is linked to aberrant cortisol levels, lower Brain Derived Neurotrophic Factor (BDNF), higher interleukin-6 (IL-6) levels, and lower amygdala and/or hippocampus volume [16]. Mesolimbic DA-ergic pathway dysfunction as well as disturbances in glutamatergic function affecting parvalbumin-positive interneurons in the cerebral cortex and hippocampus have also been discerned [271, 280]. Moreover, stress-associated signaling cascades seem to negatively impact synaptic plasticity, connectivity, and cortical micro-circuitry, further reflecting the mediating role of environmental stressors in genetically susceptible individuals to develop psychosis [271].

It must be noted that environmental adversities are not exclusively associated with psychotic spectrum disorders, but rather with multidimensional psychopathology that encompass enduring affective and behavioral problems. This fact underscores the necessity for a transdiagnostic perspective in the investigation of gene-environment interaction in mental disorders. Additionally, it is important to consider the type, timing, and extent of stressors in mental health impact, as differences imply variable modifications in emotional expression and psychosis proneness [162]. Yet another level of complexity in the interpretation of the environmental impact on stress response and early psychosis is the limitation of the study on environmental risk factors per se. The reliability of subjective experiences based on retrospective self-reports of stress and childhood traumatic incidents has been repeatedly questioned, and the possibility of reverse causation (psychosis increasing risk of environmental exposure) has been stressed [1, 16, 19]. Biological adversities, like maternal and perinatal infections, have a limited time period that might affect developmentally the offspring, while other risk factors, like cannabis use, exhibit a variable dose-dependent response [9]. Meanwhile, different environmental stressors might have a reciprocal impact, which limits their independent evaluation [274]. The particular modes of impact might also differ since some risk factors infiltrate biological pathways through gene-environment interactions, while others exert their modifications epigenetically through gene expression impacting phenomenology [16, 281]. Besides, studies addressing similar outcomes or exposure might include different samples using different psychometric tools and variables, and there is always the possibility of confounding, since not all potential environmental stressors have been identified [1, 274].

Given the environmental influence in shaping psychosis liability and precipitating expression of pre-existing vulnerability, targeting modifiable environmental factors over the lifespan provides an optimistic prospect for improving population-based mental health outcomes. Up to date, there is mounting evidence of controlled studies arguing that environmental stressors are not only genetically confounded but also have a direct impact on shaping psychosis liability [3, 282]. As in the case of genetics, various environmental factors consist of a dynamic, constantly changing, intercorrelated and interacting, multimodal, multilevel and multidimensional system, including the internal (*e.g*., immune system), the specific external (*e.g*., drug use), and the general external (*e.g*., urbanicity), and it is suggested that it should be approached as an entirety of the non-genetic component [274]. Understanding the complex and dynamic role of the environment may provide an insight into potentially modifiable factors influencing individual genetic vulnerability and optimizing treatment and precision medicine strategies. Rather than constrained in reducing symptoms, therapeutic models should be broadened in strengthening resilience in existential domains of individuals with unique mental vulnerabilities by enhancing adaptive coping mechanisms and facilitating peer support and social participation within a public health perspective [176].

## CONCLUSION AND FUTURE PROSPECTS

In the last twenty years, research on the role of the environment contributing to the emergence of psychosis has been extensive. It is evident that early adversities and psychosocial insults affect the biological, affective, and behavioral responses toward stress in genetically susceptible individuals, favoring a distorted interpretation of external reality. Stress sensitivity seems to be the key mechanism influencing psychosis risk and symptom severity. Still, despite the broad acknowledgement of gene-environment interplay in the emergence of psychosis, the obscurity concerning the pathophysiology of psychosis, along with the vast possible genetic variants and dozens of environmental determinants present in the life of individuals, impedes the complete understanding of the causal links between risk factors and expression of the disease.

Future prospects should aim at preventing potential early and late traumatic exposures to prevent the cultivation of stress sensitivity, affective dysregulation, and disturbed appraisal systems, and also to further extend research for clarifying the risk factors that lay the foundation for the complex gene-environment interaction and foster psychotic phenomena. The detection of other environmental stressors, the advancement of the evaluative measures regarding environmental risk, and the employment of aggregate scores derived from adequately powered studies could further elucidate the relationship between stress, environment, and early psychosis.

## Figures and Tables

**Fig. (2):**
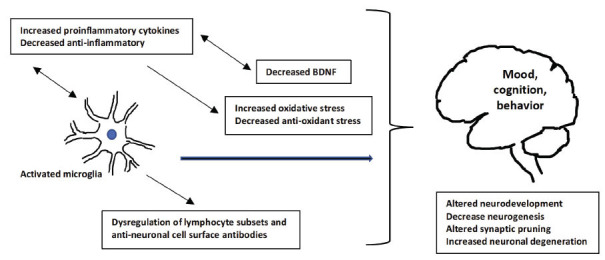
Possible mechanisms of oxidative disruption and inflammatory alterations resulting in neuroanatomical changes in early psychosis.

**Table 1:** Environmental risk factors for the emergence of psychosis.

**Early Life**	**Childhood**	**Later Life**
Parental age	Abuse	Social disadvantage and social defeat
Season of birth	Neglect	Urbanicity
Urban birth	Peer victimization	Migration and ethnical minority
Poor maternal health conditions	Disrupted familial environment	Cannabis abuse
Maternal stress	Head injury	Life events
Obstetric complications	-	-
Postnatal infections	-	-

## LIST OF ABBREVIATIONS

BDNF: Brain Derived Neurotrophic Factor
FEP: First-episode Psychosis
GR: Glucocorticoid Receptors
GWAS: Genome-wide Association Studies
HPA: Hypothalamic-pituitary-adrenal
IL-6: Interleukin-6
MR: Mendelian Randomization
RBC: Red Blood Cell
